# Supplementary material for: Elevated Biomarkers of Inflammation and Vascular Dysfunction Are Associated with Distal Sensory Polyneuropathy in People with HIV
Source: Int J Mol Sci. 2024 Apr 11;25(8):4245. doi: 10.3390/ijms25084245 (PMC11049997; doi:10.3390/ijms25084245)
Supplement: Supplementary file 1 [file ijms-25-04245-s001.zip › ijms-2859855-supplementary/Table S1.pdf]

Table S1. Pearson correlation coefficient of inflammatory and vascular integrity biomarkers.

**Biomarkers**	**MCP-1**	**TIMP-1**	**IP-10**	**IL-8**	**VEGF**	**VCAM**	**sTNFRII**	**uPAR**	**d-dimer**	**IL-6**	**MMP-2**	**TIMP-2**	**MMP-7**
**MCP-1**	1												
**TIMP-1**	**0.52**	1											
**IP-10**	**0.48**	**0.42**	1										
**IL-8**	**0.45**	**0.42**	**0.35**	1									
**VEGF**	**0.42**	**0.44**	**0.18**	**0.39**	1								
**VCAM-1**	0.06	**0.23**	**0.32**	0.07	0.07	1							
**sTNFRII**	**0.24**	**0.49**	**0.33**	**0.20**	**0.24**	**0.60**	1						
**uPAR**	0.20	**0.39**	**0.30**	0.13	0.11	**0.30**	**0.53**	1					
**d-dimer**	**0.19**	**0.29**	0.06	**0.19**	**0.19**	0.05	**0.32**	**0.50**	1				
**IL-6**	**0.31**	**0.33**	**0.31**	**0.27**	0.14	0.04	**0.37**	**0.50**	**0.44**	1			
**MMP-2**	**0.26**	**0.15**	-0.04	0.15	-0.05	0.06	0.16	0.09	0.16	0.10	1		
**TIMP-2**	0.12	**0.45**	-0.09	0.14	0.14	**0.23**	**0.30**	0.16	**0.20**	0.06	**0.42**	1	
**MMP-7**	0.13	**0.18**	0.05	**0.14**	**0.16**	**0.27**	**0.42**	0.13	0.14	**0.26**	**0.21**	0.11	1
**ICAM-1**	0.13	**0.18**	**0.22**	0.11	0.08	**0.27**	**0.35**	0.15	**0.20**	**0.22**	0.09	0.12	**0.26**

* Bold values denote significant correlation at a p-value < 0.05.
