# Supplementary material for: Elevated Biomarkers of Inflammation and Vascular Dysfunction Are Associated with Distal Sensory Polyneuropathy in People with HIV
Source: Int J Mol Sci. 2024 Apr 11;25(8):4245. doi: 10.3390/ijms25084245 (PMC11049997; doi:10.3390/ijms25084245)
Supplement: Supplementary file 1 [file ijms-25-04245-s001.zip › ijms-2859855-supplementary/Table S2.pdf]

Table S2. Association of participant characteristics and plasma biomarker factors with DSP signs and symptoms (n = 132).

	DSP*		Presence of any DSP Symptoms		DNP		Presence of Paresthesia (Tingling)		Loss of Sensation	
	OR (95%CI)	p	OR (95%CI)	p	OR (95%CI)	p	OR (95%CI)	p	OR (95%CI)	
**Univariable regression model**										
**age**	**1.11 (1.05-1.17)**	**<0.001**	**1.04 (1.01-1.07)**	**0.02**	**1.04 (1.00-1.08)**	**0.05**	1.03 (0.99-1.06)	0.08	1.01 (0.97-1.04)	0.80
**Gender (male)**	0.27 (0.06-1.27)	0.1	0.56 (0.23-1.37)	0.20	0.42 (0.11-1.56)	0.20	0.41 (0.14-1.17)	0.09	0.38 (0.11-1.42)	0.15
**Ethnicity (Hispanic)**	1.23 (0.19-8.00)	0.83	0.34 (0.07-1.74)	0.20	0.58 (0.06-5.99)	0.65	0.56 (0.10-3.07)	0.51	0.70 (0.12-3.94)	0.68
**Height**	1.12 (0.98-1.29)	0.09	1.05 (0.95-1.15)	0.35	1.05 (0.93-1.18)	0.46	**1.13 (1.01-1.27)**	**0.03**	1.08 (0.96-1.22)	0.22
**Diabetes Mellitus**	0.75 (0.08-6.75)	0.81	2.37 (0.50-11.21)	0.27	3.17 (0.56-17.91)	0.19	2.37 (0.50-11.21)	0.55	<0.001 (<0.001-)	0.99
**Metabolic Syndrome**	0.61 (0.21-1.82)	0.38	**2.76 (1.23-6.19)**	**0.01**	2.53 (0.88-7.33)	0.08	2.03 (0.85-4.84)	0.11	1.67 (0.60-4.65)	0.33
**Framingham CVD risk score**	**1.08 (1.03-1.13)**	**<0.001**	**1.06 (1.02-1.10)**	**0.003**	**1.07 (1.03-1.12)**	**0.002**	**1.05 (1.01-1.09)**	**0.02**	1.03 (0.98-1.07)	0.25
**Lifetime alcohol use disorder**	1.29 (0.42-3.95)	0.66	1.28 (0.57-2.88)	0.54	1.24 (0.43-3.56)	0.69	1.88 (0.76-4.66)	0.17	1.87 (0.65-5.41)	0.25
**Lifetime methamphetamine use disorder**	1.70 (0.33-8.69)	0.52	0.81 (0.36-1.82)	0.61	1.49 (0.51-4.28)	0.46	0.68 (0.28-1.65)	0.39	0.97 (0.35-2.71)	0.96
**Lifetime opioid use disorder**	<0.001 (<0.001-)	0.99	0.63 (0.13-3.08)	0.57	0.67 (0.08-5.59)	0.71	0.89 (0.18-4.40)	0.88	0.62 (0.07-5.17)	0.66
**HIV seropositivity**	**3.32 (1.00-11.03)**	**0.049**	**4.81 (1.97-11.73)**	**<0.001**	**1.07 (1.03-1.12)**	**0.01**	**6.15 (2.15-17.57)**	**<0.001**	**10.10 (2.21-46.23)**	**0.003**
**Factor 1**	1.66 (0.90-3.09)	0.10	**1.59 (1.01-2.53)**	**0.05**	1.77 (0.96-3.24)	0.06	1.52 (0.92-2.50)	0.1	1.16 (0.65-)	0.62
**Factor 2**	**2.01 (1.05-3.64)**	**0.02**	**2.03 (1.26-3.25)**	**0.003**	**1.82 (1.03-3.23)**	**0.04**	**2.15 (1.29-3.57)**	**0.003**	**2.54 (1.39-4.63)**	**0.002**
**Factor 3**	1.70 (0.91-3.19)	0.10	1.41 (0.89-2.23)	0.14	1.53 (0.83-2.80)	0.17	1.22 (0.75-2.01)	0.41	1.15 (0.64-2.06)	0.64
**Factor 4**	**3.56 (1.24-10.26)**	**0.02**	1.05 (0.68-1.60)	0.84	0.99 (0.57-1.71)	0.99	1.20 (0.72-1.99)	0.49	0.98 (0.58-1.67)	0.95
**Factor 5**	1.08 (0.55-2.11)	0.82	0.95 (0.58-1.54)	0.83	1.01 (0.53-1.93)	0.97	1.01 (0.65-1.88)	0.72	0.83 (0.45-1.52)	0.54
**Factor 6**	1.13 (0.56-2.23)	0.73	1.07 (0.65-1.76)	0.78	1.40 (0.69-2.83)	0.35	1.21 (0.69-2.11)	0.50	1.32 (0.67-2.60)	0.42
**Multivariable analysis****										
**Age**	**1.14 (1.06-1.23)**	**<0.001**	**1.04 (1.00-1.08)**	**0.04**	1.01 (0.95-1.07)	0.71	1.02 (0.97-1.07)	0.44	-	-
**Gender (male)**	-	-					2.89 (0.55-15.25)	0.21	-	-
**Height**	1.24 (0.93-1.64)	0.14	-	-	-	-	1.07 (0.94-1.22)	0.31	-	-
**Framingham CVD risk score**	1.07 (0.98-1.16)	0.10	1.02 (0.97-1.08)	0.42	**1.08 (1.03-1.13)**	**<0.001**	**1.05 (1.01-1.10)**	**0.02**	-	-
**Metabolic Syndrome**	-	-	**2.90 (1.11-7.54)**	**0.03**	1.41 (0.38-5.22)	0.61	-	-	-	-
**HIV seropositivity**	**6.40 (1.30-31.45)**	**0.02**	5.73 (2.02-16.23)	0.001	**7.51 (1.73-32.71)**	**0.007**	**5.24 (1.68-16.38)**	**0.004**	**7.09 (1.50-33.59)**	**0.01**
**Factor 1**	1.23 (0.41-3.78)	0.71	0.99 (0.55-1.78)	0.97	1.04 (0.48-2.25)	0.92	1.00 (0.53-1.90)	0.99	-	-
**Factor 2** (VCAM, sTNFRII)	1.98 (0.90-4.35)	0.09	1.61 (0.95-2.74)	0.09	1.33 (0.65-2.69)	0.43	1.66 (0.95-2.90)	0.08	**2.08 (1.08-3.99)**	**0.03**
**Factor 3** (uPAR, d-dimer, IL-6)	0.98 (0.32-2.70)	0.97	-	-	-	-	-	-	-	-
**Factor 4 (MMP-2)**	**5.13 (1.21-21.82)**	**0.03**	-	-	-	-	-	-	-	-

* DSP was defined as the presence of two or more DSP signs: reduced, bilateral, symmetric, distal vibration, sharp sensation, and ankle reflexes. DNP: distal neuropathic pain; DSP: distal sensory polyneuropathy; METH: methamphetamine
