# Supplementary material for: Elevated Biomarkers of Inflammation and Vascular Dysfunction Are Associated with Distal Sensory Polyneuropathy in People with HIV
Source: Int J Mol Sci. 2024 Apr 11;25(8):4245. doi: 10.3390/ijms25084245 (PMC11049997; doi:10.3390/ijms25084245)
Supplement: Supplementary file 1 [file ijms-25-04245-s001.zip › ijms-2859855-supplementary/Table S3.pdf]

Table S3. Association of demographic characteristics and plasma biomarker with DSP signs and symptoms in PWH (n = 68).

	DSP*		Presence of any DSP Symptoms		DNP		Presence of Paresthesia (Tingling)		Loss of Sensation	
	OR (95%CI)	p	OR (95%CI)	p	OR (95%CI)	p	OR (95%CI)	p	OR (95%CI)	p
**Univariable regression model**										
**Age (year)**	1.15 (1.06-1.26)	<0.001	1.04 (0.99-1.09)	0.06	1.03 (0.98-1.09)	0.17	1.03 (0.99-1.08)	0.10	1.01 (0.96-1.05)	0.73
**Gender (male)**	0.96 (0.10-9.13)	0.97	0.76 (0.13-4.50)	0.76	0.77 (0.09-7.19)	0.82	1.03 (0.17-6.11)	0.98	0.63 (0.07-5.84)	0.68
**Ethnicity (Hispanic)**	1.58 (0.12-20.7)	0.73	0.42 (0.04-4.24)	0.46	1.00 (0.08-11.5)	0.99	0.71 (0.07-7.66)	0.77	0.71 (0.06-7.66)	0.77
**Height**	1.04 (0.86-1.24)	0.69	1.05 (0.91-1.21)	0.50	1.03 (0.87-1.22)	0.74	1.11 (0.95-1.30)	0.18	1.07 (0.91-1.26)	0.44
**Diabetes Mellitus**	5.20 (0.30-90.2)	0.26	<0.001 (<0.001-)	0.99	<0.001 (<0.001-)	0.99	<0.001 (<0.001-)	0.99	<0.001 (<0.001)	0.99
**Metabolic Syndrome**	1.92 (0.51-7.24)	0.33	3.08 (1.04-9.08)	0.04	2.06 (0.59-7.15)	0.25	2.64 (0.88-7.92)	0.08	1.51 (0.46-5.01)	0.50
**Framingham CVD risk score**	1.13 (1.05-1.22)	0.001	1.06 (1.01-1.13)	0.03	1.07 (1.01-1.14)	0.03	1.05 (0.99-1.11)	0.09	1.03 (0.97-1.09)	0.29
**Duration of ART**	1.00 (0.99-1.01)	0.53	1.01 (0.99-1.01)	0.17	1.01 (0.99-1.01)	0.21	1.00 (0.99-1.01)	0.45	1.01 (0.99-1.01)	0.51
**Prior exposure to the D-drugs**	3.70 (0.54-25.38)	0.18	2.52 (0.39-16.29)	0.33	0.98 (0.10-9.58)	0.98	3.42 (0.52-22.23)	0.20	5.87 (0.88-39.20)	0.07
**Duration of infection**	1.10 (1.02-1.19)	0.01	1.05 (0.99-1.11)	0.12	1.05 (0.98-1.13)	0.14	1.05 (0.99-1.11)	0.13	1.05 (0.98-1.12)	0.14
**Plasma viral load**	1.17 (0.53-2.57)	0.70	0.83 (0.42-1.65)	0.60	1.03 (0.47-2.25)	0.94	0.56 (0.23-1.35)	0.20	0.49 (0.16-1.45)	0.20
**Current CD4 T-cells count**	1.00 (0.99-1.00)	0.75	0.99 (0.99-1.00)	0.35	1.00 (0.99-1.00)	0.74	1.00 (0.99-1.00)	0.72	1.00 (1.00-1.00)	0.09
**Lifetime alcohol use disorder**	1.63 (0.38-7.05)	0.51	1.14 (0.40-3.27)	0.81	1.05 (0.30-3.69)	0.94	1.82 (0.58-5.70)	0.30	1.41 (0.41-4.78)	0.58
**Lifetime methamphetamine use disorder**	0.33 (0.08-1.34)	0.12	0.42 (0.15-1.23)	0.11	0.96 (0.27-3.39)	0.95	0.24 (0.08-0.76)	0.02	0.61 (0.19-1.99)	0.41
**Lifetime opioid use disorder**	<0.001 (<0.001-)	0.99	0.49 (0.05-5.03)	0.55	1.25 (0.12-13.12)	0.85	0.66 (0.07-6.85)	0.73	1.02 (0.10-10.7)	0.98
**Factor 1**	1.52 (0.70-3.31)	0.29	1.48 (0.80-2.73)	0.21	1.43 (0.69-2.97)	0.33	1.23 (0.67-2.28)	0.51	0.90 (0.46-1.76)	0.76
**Factor 2**	3.12 (1.27-7.70)	0.01	1.58 (0.84-2.96)	0.16	1.42 (0.68-2.95)	0.35	1.46 (0.77-2.77)	0.25	2.30 (1.07-4.94)	0.03
**Factor 3**	2.25 (1.02-4.98)	0.04	1.47 (0.81-2.70)	0.21	1.57 (0.77-3.20)	0.22	1.43 (0.77-2.65)	0.26	1.21 (0.61-2.37)	0.58
**Factor 4**	2.40 (0.77-7.49)	0.13	1.07 (0.60-1.90)	0.83	0.92 (0.47-1.79)	0.81	1.12 (0.61-2.07)	0.71	1.10 (0.55-2.17)	0.78
**Factor 5**	1.19 (0.56-2.51)	0.65	1.36 (0.76-2.43)	0.30	1.41 (0.68-2.93)	0.35	1.33 (0.73-2.44)	0.35	1.03 (0.54-1.97)	0.92
**Factor 6**	0.83 (0.39-1.78)	0.64	0.90 (0.47-1.67)	0.74	1.25 (0.55-2.81)	0.59	1.01 (0.53-1.93)	0.97	1.66 (0.71-3.89)	0.24
**Multivariable logistic regression model***										
**Age**	1.18 (1.07-1.30)	<0.001	1.02 (0.96-1.09)	0.57	-	-	1.01 (0.95-1.08)	0.76	-	-
**Lifetime methamphetamine use disorder**	-	-	-	-	-	-	3.82 (1.23-11.87)	0.02	-	-
**Framingham CVD risk score**	0.97 (0.85-1.10)	0.61	1.07 (1.01-1.13)	0.03	1.07 (1.01-1.14)	0.03	1.02 (0.92-1.12)	0.76	-	-
**Metabolic Syndrome**	-	-	1.85 (0.54-6.35)	0.33	-	-	1.58 (0.42-5.95)	0.50	-	-
**Duration of infection**	1.05 (0.95-1.17)	0.35	-	-	-	-	-	-	-	-
**Prior exposure to the D-drugs**	-	-	-	-	-	-	-	-	9.43 (1.23-72.10)	0.03
**Current CD4 T-cells count**	-	-	-	-	-	-	-	-	0.99 (0.99-1.00)	0.08

**Factor 1**	-	-	-	-	-	-	-	-	-	-
**Factor 2** (VCAM, sTNFRII)	5.45 (1.42-21.0)	0.01	-	-	-	-	-	-	2.30 (1.08-4.91)	0.03
**Factor 3** (uPAR, d-dimer, IL-6)	0.54 (0.16-1.85)	0.33	-	-	-	-	-	-	-	-
**Factor 4** (MMP-2)	-	-	-	-	-	-	-	-	-	-

* DSP was defined as the presence of two or more of the following DSP signs: reduced, bilateral, symmetric, distal vibration, sharp sensation, and ankle reflexes.
DNP: distal neuropathic pain.
