# Supplementary material for: Elevated Biomarkers of Inflammation and Vascular Dysfunction Are Associated with Distal Sensory Polyneuropathy in People with HIV
Source: Int J Mol Sci. 2024 Apr 11;25(8):4245. doi: 10.3390/ijms25084245 (PMC11049997; doi:10.3390/ijms25084245)
Supplement: Supplementary file 1 [file ijms-25-04245-s001.zip › ijms-2859855-supplementary/Table S4.pdf]

Table S4. Association of demographic characteristics and plasma biomarker with DSP signs and symptoms in PWoH (n = 64).

	DSP*		Presence of any DSP Symptoms		DNP		Presence of Paresthesia (Tingling)		Loss of Sensation	
	**OR (95%CI)**	**p**	**OR (95%CI)**	**p**	**OR (95%CI)**	**p**	**OR (95%CI)**	**p**	**OR (95%CI)**	**p**
**Univariable analysis**										
**age (year)**	1.11 (1.00-1.24)	0.07	1.06 (1.00-1.13)	0.047	1.16 (0.99-1.37)	0.07	ns	ns	ns	ns
**Gender (male)**	ns	ns	ns	ns	ns	ns	ns	ns	ns	ns
**Ethnicity (Hispanic)**	ns	ns	ns	ns	ns	ns	ns	ns	ns	ns
**Height**	ns	ns	ns	ns	ns	ns	ns	ns	ns	ns
**Diabetes Mellitus**	ns	ns	17.4 (2.33-129)	0.005	41.33 (2.87-594)	0.01	13.33 (1.58-112)	0.02	ns	ns
**Metabolic Syndrome**	ns	ns	5.57 (1.03-30.1)	0.046	ns	ns	ns	ns	ns	ns
**Framingham CVD risk score**	ns	ns	1.06 (1.01-1.13)	0.03	1.10 (1.02-1.18)	0.01	1.06 (0.99-1.12)	0.06	ns	ns
**Lifetime alcohol use disorder**	ns	ns	ns	ns	ns	ns	ns	ns	ns	ns
**Lifetime methamphetamine use disorder**	ns	ns	ns	ns	ns	ns	ns	ns	ns	ns
**Lifetime opioid use disorder**	ns	ns	ns	ns	ns	ns	ns	ns	ns	ns
**Factor 1**	ns	ns	ns	ns	ns	ns	ns	ns	ns	ns
**Factor 2**	ns	ns	ns	ns	ns	ns	2.57 (0.96-6.93)	0.06	ns	ns
**Factor 3**	ns	ns	3.01 (1.08-8.42)	0.03	4.81 (0.84-27.4)	0.08	ns	ns	ns	ns
**Factor 4**	15.2 (1.07-215)	0.04	ns	ns	ns	ns	ns	ns	ns	ns
**Factor 5**	ns	ns	ns	ns	ns	ns	ns	ns	ns	ns
**Factor 6**	ns	ns	ns	ns	ns	ns	3.96 (0.91-14.2)	0.07	ns	ns
**Multivariable analysis***										
**Diabetes mellitus**			13.2 (1.71-102)	0.01	ns	ns	ns	ns		
**Factor 2** (VCAM, sTNFRII)	-	-	-	-	-	-	3.79 (0.98-14.6)	0.053	-	-
**Factor 4** (MMP-2)	49.24 (1.26-1921)	0.04	-	-	-	-	-	-	-	-

* DSP was defined as the presence of two or more of the following DSP signs: reduced, bilateral, symmetric, distal vibration, sharp sensation, and ankle reflexes. DNP: distal neuropathic pain.
